# pH-Sensitive Mebeverine Microspheres for Colon Delivery

**DOI:** 10.4103/0250-474X.57303

**Published:** 2009

**Authors:** P. M. Dandagi, V. S. Mastiholimath, A. P. Gadad, A. R. Kulkarni, B. K. Konnur

**Affiliations:** Department of Pharmaceutics, K. L. E. S's College of Pharmacy, J. N. M. C. Campus, Nehru Nagar, Belgaum-590 010, India; 1 Department of Pharmacology, K. L. E. S's College of Pharmacy, J. N. M. C. Campus, Nehru Nagar, Belgaum-590 010, India

**Keywords:** pH-sensitive microspheres, mebeverine hydrochloride, emulsion solvent evaporation technique, Eudragit S100, Eudragit L100

## Abstract

Mebeverine hydrochloride is known to suffer from extensive first pass effect. In an attempt to improve its oral bioavailability and possibility to restrict its absorption only to the colon, mebeverine microspheres were prepared by emulsion solvent evaporation method. Four formulations were prepared with varying drug and polymer ratio. These formulations were subjected to various evaluation parameters like percent practical yield, entrapment efficiency, particle size, *in vitro* drug release, *in vivo* activity. Practical yield of the microspheres was up to 89.59% with encapsulation efficiency up to 79.4%. Scanning electron microscopy confirmed that the microsphere structures were smooth, spherical, and discrete and the particles were of the size range 200 to 300 μm. *In vitro* release of the drug showed biphasic release pattern with non-Fickian diffusion release in 12 h. On the basis of drug content, particle size, *in vitro* release and *in vivo* studies, formulation F-3 was found to be optimal. Antiirritable bowel syndrome activity was performed in colorectal distention in rat, which is a model for constipation-induced irritable bowel syndrome. The formulations F-2 and F-3 showed significant effect in fecal output when compared to the control as well as the marketed preparation in the constipation-induced irritable bowel syndrome in rats.

Mebeverine hydrochloride (MBH) is having poor oral bioavailability and has some adverse effects, even though it shows direct action on the smooth muscle of the GIT (especially colon), because of this reason the mebeverine hydrochloride is used as model drug for site-specific drug delivery system[1,2]. Most of the conventional drug delivery systems for treating the colon disorders such as inflammatory bowel diseases (e.g. irritable bowel syndrome, ulcerative colitis and Crohn's disease), infectious diseases (e.g. amoebiasis) and colon cancer are failing as the drugs do not reach the site of action in appropriate concentrations[3].

Rectal dosage forms such as suppositories and enemas are not always effective since a high variability in the distribution of these dosage forms is observed. Suppositories are only effective in the rectum because of the confined spread and enema solutions can only offer topical treatment to the sigmoid and descending colon. Suppositories are melting or soften in the rectum and may give a feeling of alien or discomfort to the patients[3]. These limitations can be overcome by targeting drug to the colonic region. Thus, an effective therapy of the colonic disorders can be effectively achieved by using site-specific pH-sensitive drug delivery system. The present studies are aimed to targeting the colon by using pH-sensitive system and protect the active drug against first pass effect and gastrointestinal disturbances. Advantages of targeting drug to the diseased organ (colon) includes, delivery of drug in its intact form as close as possible to the target site, ability to reduce the conventional dose, reduce incidence of adverse side effects and avoidance of mucosal metabolism[3].

Mebeverine hydrochloride was supplied as a gift sample by Rantus Pharma Pvt. Ltd Hyderabad, India. Eudragit L-100 and S-100 were supplied by Degussa India Pvt.Ltd., Mumbai, India. Liquid paraffin was procured from Ranbaxy Fine Chemicals Ltd., New Delhi, India, Span 80 from Research Laboratories, Mumbai, India and analytical grade ethanol, acetone, petroleum ether from S. D. Fine Chem. Ltd., Mumbai, India.

Accurately weighed Eudragit L-100 and S-100 in 1:2 ratios were dissolved in 10 ml of acetone and 5 ml of alcohol to form a homogenous polymers solution. Mebeverine hydrochloride was dispersed in it and mixed thoroughly. This organic phase was slowly poured at 30° in to liquid paraffin (100 ml) containing 1% (w/w) of Span-80 with stirring at 800 rpm to form a uniform emulsion[4,5]. Thereafter, it was allowed to attain room temperature and stirring was continued until residual acetone evaporated and smooth-walled, rigid and discrete microspheres were formed. The microspheres were collected by decantation and the product was washed with petroleum ether or n-hexane (40-60^°^), four times and dried at room temperature for 3 h. The microspheres were then stored in a dessicator over fused calcium chloride. Four batches of formulation were prepared with different proportions of core to coat materials as shown in Table 1 and labeled as F-1, F-2, F-3 and F-4, respectively.

**TABLE 1:** FORMULATIONS WITH CORE:COAT RATIO, PERCENTAGE YIELD, PERCENTAGE ENTRAPMENT EFFICIENCY AND PARTICLE SIZE

Formulations	Core:Coat ratio	Percentage Yield (%)	Entrapment Efficiency (%)	Particle Size (μm)#
F-1	1:0.5	79.40	63.2	224 ± 4.15
F-2	1:1	83.76	68.9	240 ± 6.75
F-3	1:1.5	88.33	79.4	245 ± 4.25
F-4	1:2	89.59	78.8	259 ± 7.55

Values expressed as average of triplicates,

# Arithmetic mean of 100 particles expressed as mean±SEM

Average particle size determination of mebeverine hydrochloride microspheres of all the formulations was carried out by optical microscopy using calibrated ocular micrometer. Scanning electron microscopy was carried out using Jeol-JSM T-330A scanning electron microscope, Japan to study surface topography of microspheres. Dry microspheres were placed on an electron microscope brass stub and coated with gold in an ion sputter. Photographs of microspheres were taken by random scanning of the stub[5].

Drug content was estimated by taking 50 mg of crushed microspheres in 100 ml volumetric flask containing pH 7.4-phosphate buffer. The flasks were shaken for 12 h using an orbital shaker incubator. Then the solution was filtered and from the filtrate, appropriate dilutions were made and absorbance was measured at 262 nm using UV spectrophotometer[6].

The drug dissolution tests of microspheres were carried out by the paddle method specified in the US Pharmacopoeia XXI. Microspheres were weighed (weight equivalent to 50 mg of drug were filled in tea bags. The tea bag tied with paddle and immersed in 900 ml of dissolution medium and rotated at 100 rpm at 37^°^. Perfect sink conditions prevailed during the dissolution tests. The sample aliquot were withdrawn at an appropriate interval from the dissolution vessel and assayed spectrophotometrically at 262 nm[4,5,7].

Colorectal distention in rat model is used for *in vivo* studies. This model produced constipation predominant irritable bowel syndrome (C-IBS), prior approval is obtained from the institutional animal ethics committee to conduct the study. The rat model was set up by intragastric instillation of 2.0 ml water at 0-4° in male Wistar rats for 2 w. All the groups underwent rectal distention except normal control group under deep anesthesia. All the rats were allowed free access to food and water throughout the experiment[8]. Male rats weighing around 200 g were used for the experiment. Animals were divided in to five groups each groups with six animals, group one normal control underwent without C-IBS induction, group two is negative control, underwent C-IBS induction but no treatment, group three received the marketed product while group four and five were treated with formulated microspheres. After treatment, animals were placed in individual wire-bottomed cages and fecal output was monitored at 4, 8, 12, and 24 h. The fecal pellet output in all the four groups was used as an index of colonic motility and transit[9].

To ensure the safety, efficacy and quality of active drug substance and dosage forms stability studies were performed as per OECD guidelines. Prepared formulations were divided in to 3 sample sets stored at 4° in refrigerator, 37±2°/65±5% RH in humidity control oven (Ginkya IM 3500 series) and ambient temperature and humidity. At regular interval and after 30 days drug content of the formulations was determined by the method discussed previously. Changes in drug content and release behavior are observed to ensure stability of the drug and formulation.

The aim of the present study is to design pH-sensitive microspheres for colonic drug delivery for the improved treatment of irritable bowel syndrome. To prepare pH-dependent microspheres emulsion solvent evaporation technique was used as it yields consistently uniform sized microspheres. Petroleum ether or n-hexane was used to wash the microspheres since it removes liquid paraffin without affecting the integrity of the microspheres. Overall four formulations with different core: coat ratios were prepared and it was found that smooth walled, rigid and discrete microspheres were obtained.

Particle size analysis by optical microscope indicated the presence of almost uniform sized microspheres. The average mean particle sizes of the microspheres were found to be uniform and consistent (Table 1). The average mean particle size of the microspheres increased significantly with increase in polymer concentration due to high viscosity of medium at a higher polymer concentration resulting in enhanced interfacial tension and diminished shearing efficiency. Scanning electron microscope studies shows particles of F-1 and F-2 were rough surfaced and crystals of the drug are visible on surface, indicating the concentration of polymeric solution is insufficient for complete encapsulation. The F-3 and F-4 were found to be spherical, smooth and discrete. Smoothness of microspheres increases with increasing the polymer concentration, which is observed from scanning electron photomicrographs of four formulations (fig. 1). The drug content was found to be high in all the cases probably due to polymer loss by adherence to the container as a result of viscous nature of slurry. It was observed that entrapment efficiency increased with increase in concentration of polymer added in formulations (Table 1).

**Fig. 1:**
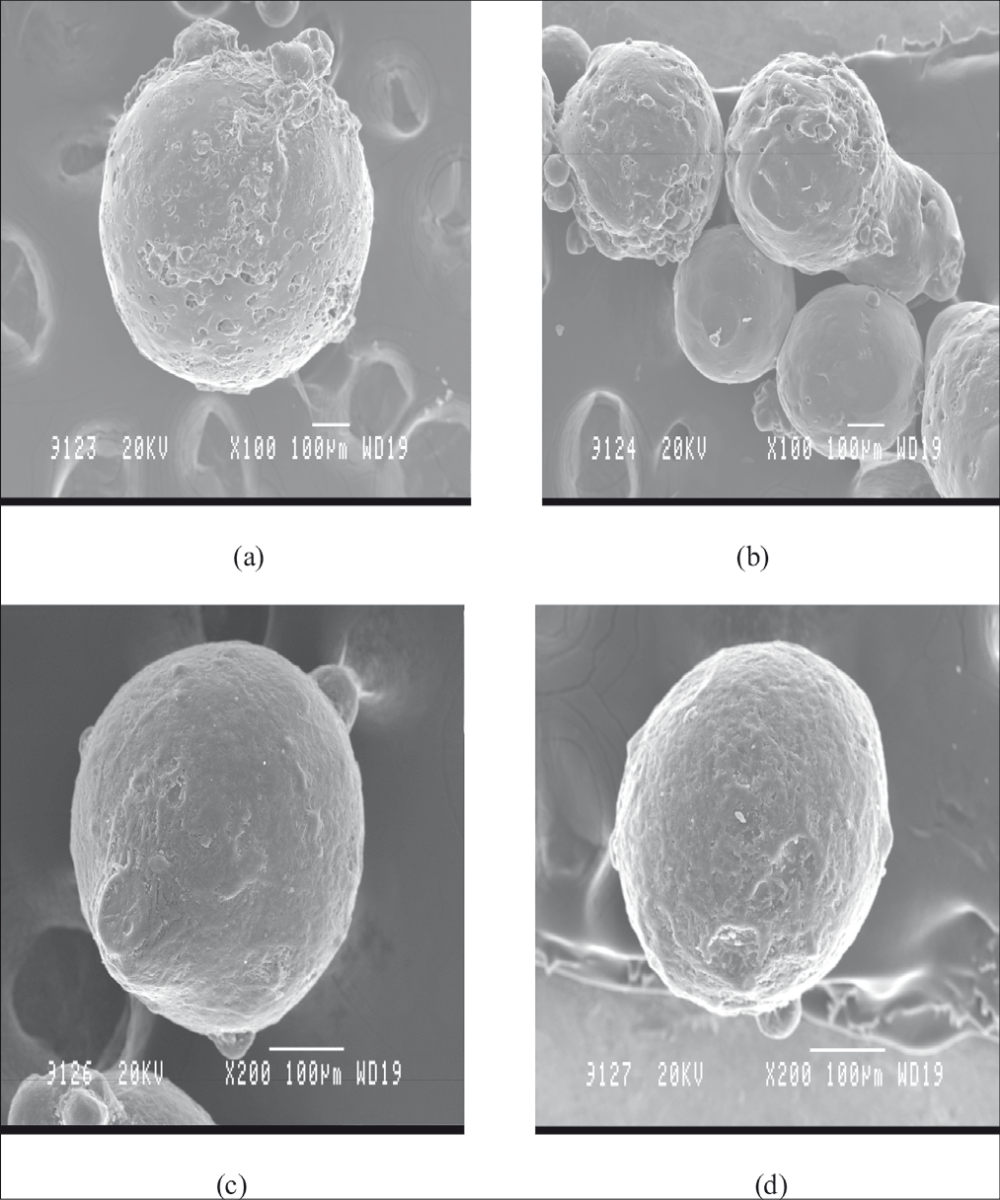
SEM Images of different formulations of mebeverine hydrochloride microspheres SEM Images Showing (a) Formulation (F-1) with core and coat ratios of 1:0.5; (b) Formulation (F-2) with 1:1; (c) Formulation (F-3) with 1:1.5 and (d) formulation (F-4) with 1:2.

*In vitro* studies were carried out using USP XXIII dissolution assembly. The release profile obtained for all the four formulations were shown in (fig. 2). It was observed that the drug release from the formulations decreased with increase in the amount of polymer added in each formulation. The release of drug from polymer matrix takes place after complete swelling of the polymer and as the amount of polymer in the formulation increase the time required to swell also increase there by decrease in the drug release. However, the release showed a bi-phasic release pattern with an initial burst effect. The mechanism for the burst release can be attributed to the drug loaded on the microspheres or imperfect or incomplete entrapment of drug.*In vitro* release data was further analyzed using various mathematical models. Overall, the curve fitting into various mathematical models was found to be average. Based on highest regression value (*r*) the best-fit model for F-1 and F-4 was Peppas whereas F-2 and F-3 found to follow Higuchi matrix, indicating that the release is by diffusion mechanism from these formulations. The ‘*n’* value obtained from Peppas model for F-1 was 0.3538, which is less than 0.45. This indicates that the release mechanism is by Fickian diffusion and formulations F-2, F-3 and F-4 exhibited ‘n’ values more than 0.45, indicating the release mechanism is non Fickian diffusion.

**Fig. 2:**
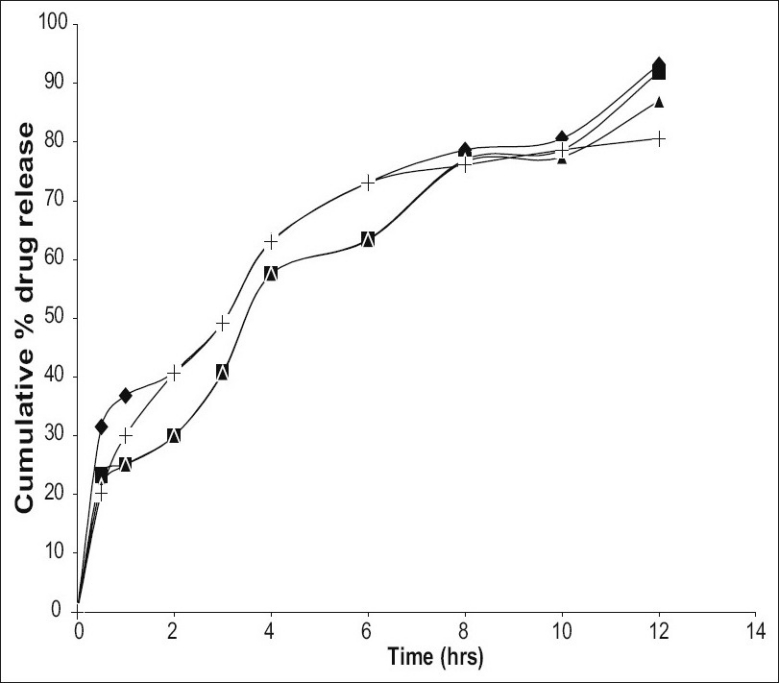
Plots of *in vitro* cumulative percentage drug released vs. time for different formulations of mebeverine hydrochloride microspheres. (–♦–) F-1 with core:coat ratio of (1:0.5); (–▪–) F-2 with (1:1); (–▲–) F-3 with (1:1.5) and (–+–) F-4 with (1:2)

Two formulations F-2 and F-3 showing promising release behavior were selected for screening antiirritable bowel syndrome activity. Screening activities of test groups (F-2 and F-3) were compared with marketed preparation (conventional) of mebeverine hydrochloride tablet. Statistical analysis was done by ANOVA (One way analysis of variance) followed by Dunnet's ‘t’ test was performed. The results of fecal output after 24 h in all the groups after treatment are represented in fig. 3. It is evident that the formulations F-2 and F-3 treated groups showed significant effect in fecal output when compared to the negative control, however better than marketed product. This shows that the mebeverine hydrochloride formulated microspheres were significantly effective in treating the constipation predominant irritable bowel syndrome (C-IBS) in rat model than the marketed preparation. Formulated microspheres were found to be stable and compatible at tested temperature and Humidity. From the study it can be concluded that Eudragit polymers (Eudragit S100 and Eudragit L100) are suitable and biocompatible polymer for the preparation of mebeverine-loaded microspheres.

**Fig. 3:**
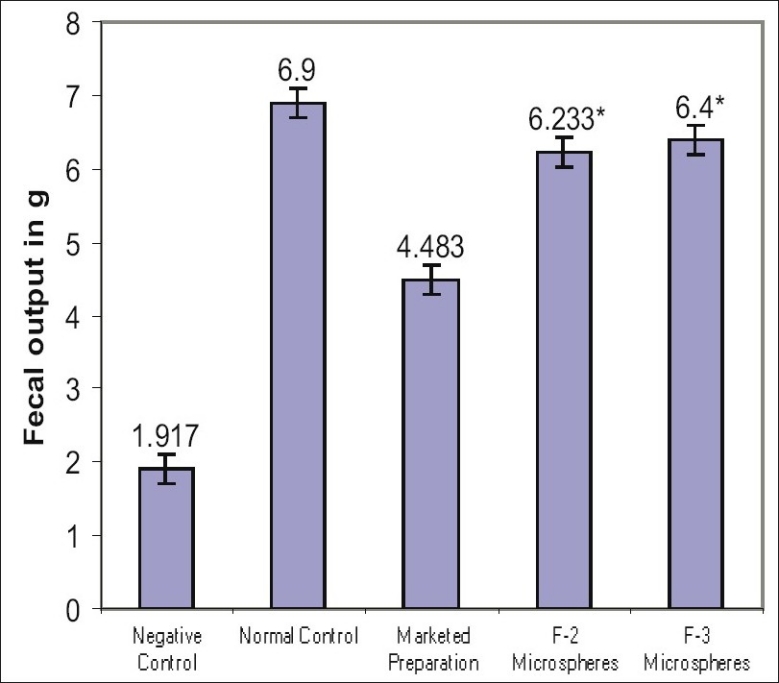
Chart depicting mean fecal output after 24 h in treated and control groups. Percentage fecal output after 24 h. Values expressed as mean±SEM, (n = 6), *P<0.001 and Dunnet's ‘t’ test * *P*<0.001
